# Discrimination of Juvenile Yellowfin (*Thunnus albacares*) and Bigeye (*T. obesus*) Tunas using Mitochondrial DNA Control Region and Liver Morphology

**DOI:** 10.1371/journal.pone.0035604

**Published:** 2012-04-19

**Authors:** Ivane R. Pedrosa-Gerasmio, Ricardo P. Babaran, Mudjekeewis D. Santos

**Affiliations:** 1 College of Fisheries and Ocean Sciences, University of the Philippines Visayas, Iloilo, Philippines; 2 National Fisheries Research and Development Institute, Quezon City, Philippines; Biodiversity Insitute of Ontario - University of Guelph, Canada

## Abstract

Yellowfin tuna, *Thunnus albacares* (Bonnaterre, 1788) and bigeye tuna, *Thunnus obesus* (Lowe, 1839) are two of the most economically important tuna species in the world. However, identification of their juveniles, especially at sizes less than 40 cm, is very difficult, often leading to misidentification and miscalculation of their catch estimates. Here, we applied the mitochondrial DNA control region D-loop, a recently validated genetic marker used for identifying tuna species (Genus *Thunnus*), to discriminate juvenile tunas caught by purse seine and ringnet sets around fish aggregating devices (FADs) off the Southern Iloilo Peninsula in Central Philippines. We checked individual identifications using the Neighbor-Joining Method and compared results with morphometric analyses and the liver phenotype. We tested 48 specimens ranging from 13 to 31 cm fork length. Morpho-meristic analyses suggested that 12 specimens (25%) were bigeye tuna and 36 specimens (75%) were yellowfin tuna. In contrast, the genetic and liver analyses both showed that 5 specimens (10%) were bigeye tuna and 43 (90%) yellowfin tuna. This suggests that misidentification can occur even with highly stringent morpho-meristic characters and that the mtDNA control region and liver phenotype are excellent markers to discriminate juveniles of yellowfin and bigeye tunas.

## Introduction

Yellowfin tuna (*Thunnus albacares*) and bigeye tuna (*T. obesus*) are the second and third most important large tuna commodity in the Philippines by catch weight, after skipjack tuna [1], [2], [3]. Following the introduction of fish aggregating devices (FADs), locally known as payao, their catches increased significantly in the mid 1970s [4] especially for smaller-sized individuals [1]. Reliable estimates of the numbers of these two species are very important in fisheries management to illustrate annual production, demonstrate utilization rates, monitor catch quotas, estimate fishing mortality and to calculate catch per unit effort especially in light of declining population due to overfishing in recent years [2]. However, differentiation of these tuna species in commercial landings poses a problem since the two species are morphologically very similar, especially at sizes less than 40 cm Fork Length (FL). It has been suggested that misidentification by fishery-data collectors can be as high as 30% (Chow and Inoue, 1993). The difficulty in distinguishing these two species, particularly to non-expert field staff has long been problematic in Philippine fisheries statistics as yellowfin and bigeye tuna data were collectively grouped as yellowfin [5].

Mis-identification is not uncommon for yellowfin and bigeye tuna species [6], with the frequency of misidentification as high as 30% [7]. Grewe and Hampton (1998) reported a 0–10.4% frequency of yellowfin among collected bigeye tunas at sizes 40 to 60 cm FL indicating the need for individual genetic identification. Interestingly, in the genetic component of the same study up to 30% of juvenile fish identified in the field as yellowfin tuna were genetically confirmed to be bigeye tuna [8].

Recently, the mitochondrial DNA control region (CR) has been validated as a molecular marker for differentiating *Thunnus* species, and is a more robust marker, than the standard mitochondrial DNA barcode marker, CO1, for differentiating all tuna species including those belonging to the subgenus *Neothunnus* (*Thunnus albacares*, *T. atlanticus*, *T. tonggol*) that are very closely related [9]. In tunas, the mtDNA CR displacement-loop (D-loop) region is highly polymorphic [10]. This does not encode proteins and typically have a high mutation rate presumably due to reduced functional constraints and relaxed selection pressure [11], increasing their likelihood of discriminating between species. In the study of Niwa *et al.* (2003), the genetic variation of the yellowfin tuna mtDNA CR D-loop was shown to be extremely high and a suitable region for investigations of population structure [10].

Here, we applied the mtDNA CR D-loop as a marker to differentiate juvenile yellowfin and bigeye tunas, and compared molecular results against identifications based on traditional characters. In addition, the liver phenotypes of the two species [12] were validated for identification of juvenile yellowfin and bigeye tuna. The right, medial and left lobes of the liver measured were analyzed using Principal Component Analysis (PCA).

## Results

### Species identification based on morpho-meristic characters

External characters such as body coloration, marks and bandings, eye diameter, and body depth have been used to identify and distinguish tunas. Body color is ideal when the specimens are fresh, but colors fade quickly after death. Similarly, bandings and lines can become washed or rubbed out. In this study, the specimens were taken from the landed catch and were stored on ice to maintain colors, markings and bands until examination. Eye [12] diameter and body depth have been used to distinguish the two species but are unreliable in juvenile specimens, since the eye of yellowfin tuna juveniles may appear quite large and indistinguishable from that of bigeye tuna . Body depths are also very similar in juveniles of both species. Takeyama *et al.* (2001) have claimed that there are no external morphological characters for species identification of small juvenile tunas.

In this study, the number of gill rakers in the lower gill arch varied between juveniles (13–31 cm FL) with 18–22 in yellowfin tuna and 17–21 in bigeye tuna and this character did not provide identification since the ranges overlap.

Using the combined traditional morpho-meristic characters above, 36 juvenile specimens were tentatively identified as yellowfin tuna and 12 specimens as bigeye tuna. These initial identifications were recorded to test the commonality of misidentification of juvenile yellowfin and bigeye tuna.

### Species identification based on mtDNA CR D-Loop

Reference control region sequences (397 base pairs) were extracted from the study of Martinez *et al.* (2006, [13]) for *T. albacares* (GenBank Accession Number DQ126342 and DQ126343) and *T. obesus* (GenBank Accession Number DQ126501 and DQ126502). Percent homology or percent identity between *T. albacares* and *T. obesus* was 90% [14].

**Figure 1:**
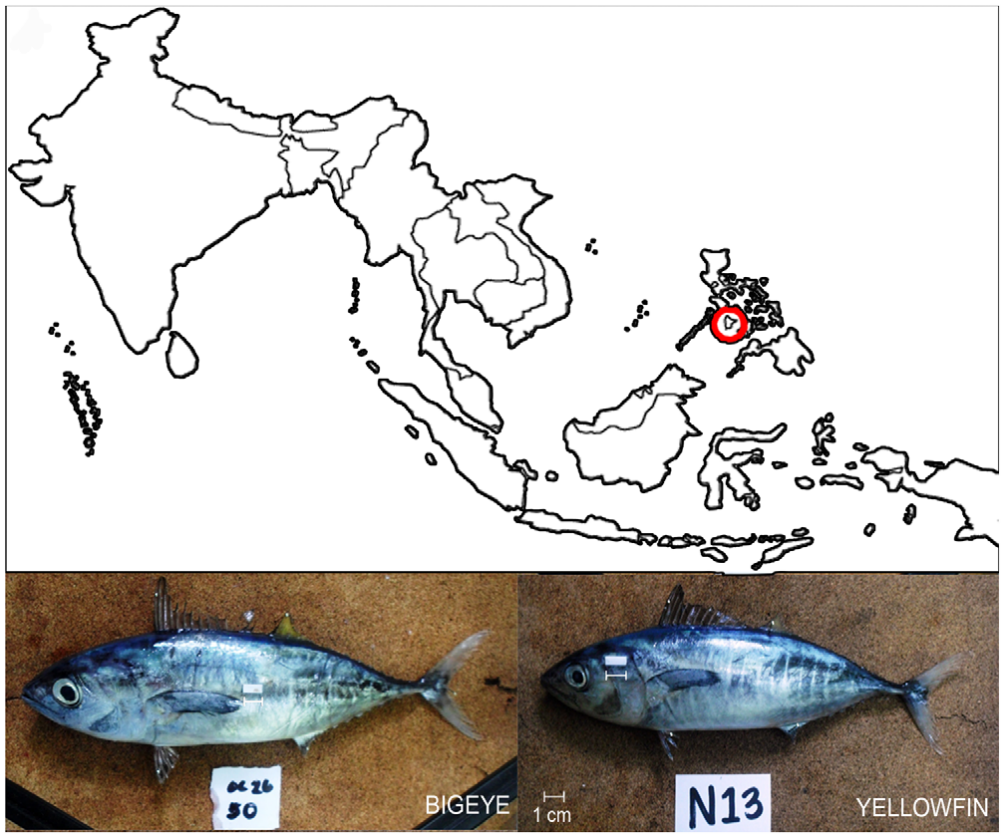
Neighbor-Joining Analyses of a 298 base pair fragment of the mtDNA CR D-loop. Numbers above the branches indicate bootstrap values inferred from 1000 replicates. Branches corresponding to partitions reproduced in less than 50% bootstrap replicates were collapsed. The tree is drawn to scale, with branch lengths in the same units as those of the evolutionary distances used to infer the phylogenetic tree.

Of the 48 DNA samples of juvenile tuna examined, Neighbor-Joining analysis identified 43 as yellowfin tuna (90%) and five as bigeye tuna (10%) (Fig. 1). Additional tree building methods (UPGMA, Minimum Evolution, and Maximum Parsimony) generated similar trees.

### Species identification based on liver morphology

**Figure 2:**
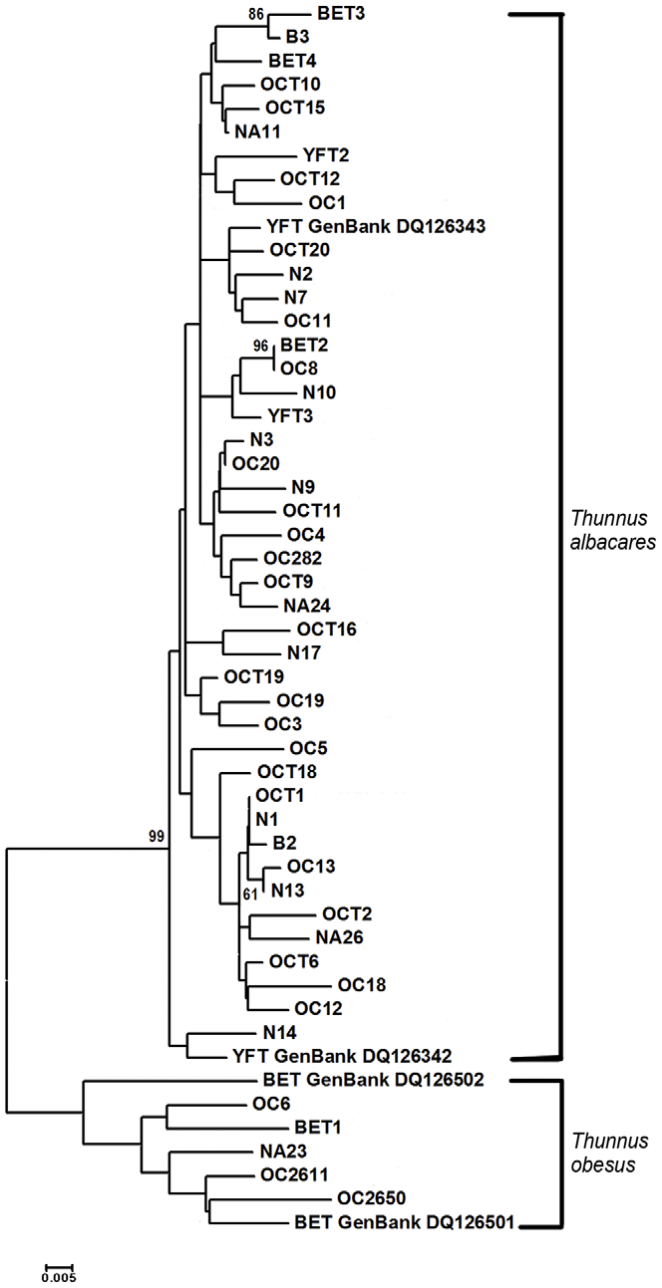
Graph Ordination from Principal Component Analysis. Principal Components I and II separated juveniles of yellowfin (Upper; n = 43) and bigeye (Lower; n = 5) tunas and comparison of their liver morphologies; yellowfin (A. Itano, 2005 and B. Pedrosa-Gerasmio *et al.*, 2011) and bigeye tuna (C. Itano, 2005 and D. Pedrosa-Gerasmio *et al.*, 2011).

Whole livers obtained from the 48 specimens showed two distinct phenotypes that corresponded with yellowfin tuna and bigeye tuna (Fig. 2). In yellowfin tuna, the right lobe of the liver is longer than the round medial and left lobes, and the lobes are smooth and clear, with no striations. In bigeye tuna, the three lobes are rounded and about equal in size, with a striated ventral surface [12]. The relative size of the right lobe provided an unambiguous separation between the juvenile specimens, but the striations were not obvious.

Principal Component Analysis (PCA) generated a graph ordination (Fig. 2) clearly separating the two species of juvenile tunas based on liver measurement data. The first two eigenvalues are approximately 95% of the total meaningful variance. In general, once eigenvectors are found from the covariance matrix, the next step is to sort them by eigenvalue, in decreasing order which gives the components (Tables 1 and 2) in order of significance [15]. In this case, the first and second components (PCI and PCII) were retained for the analysis, which yielded two axes. Genetic-based identification and liver morphology identification were in complete agreement for all individuals (Table 3).

**Table 1:** Variance extracted from the 3 axes using liver measurement data.

Axis	Eigenvalue	% of Variance	Cum.% of Var.
1	2.42	80.654	80.654
2	0.443	14.772	95.426
3	0.137	4.574	100

**Table 2:** Loadings from a Principal Component Analysis of the log transformed right, middle and left lobe measurements of *T. albacares* and *T. obesus*. Variables with the highest values on principal components I and II (in asterisks) are shown.

	EIGENVECTORS		
Characters	1	2	3
RIGHT	−0.531	0.8455*	−0.054
MIDDLE	−0.6026*	−0.333	0.7254

**Table 3:** Identification of juvenile tunas caught in Southern Iloilo, Philippines based on body morphology, liver morphology and mtDNA Control Region sequence Data (n = 48).

SAMPLE	FORK	BODY MORPHOLOGY	LIVER	CR D-LOOP	GENBANK
NAME	LENGTH (cm)	INITIAL ID	MORPHOLOGY	ID	ACCESSION
			ID		NUMBER
1. Oc6	12.8	Bigeye	Bigeye	Bigeye	JN988649
2. Oct11	21.4	Yellowfin	Yellowfin	Yellowfin	JN988644
3. Oct9	21.1	Yellowfin	Yellowfin	Yellowfin	JN988643
4. Oc4	16.4	Yellowfin	Yellowfin	Yellowfin	JN988642
5. BET3	31.6	Bigeye*	Yellowfin	Yellowfin	JN988641
6. Oc13	14.8	Yellowfin	Yellowfin	Yellowfin	JN988640
7. Oct6	22.4	Yellowfin	Yellowfin	Yellowfin	JN988639
8. BET2	31.3	Bigeye*	Yellowfin	Yellowfin	JN988638
9. BET4	28.8	Bigeye*	Yellowfin	Yellowfin	JN988637
10. B3	30	Bigeye*	Yellowfin	Yellowfin	JN988636
11. YFT2	30.4	Yellowfin	Yellowfin	Yellowfin	JN988635
12. Oc18	13.7	Yellowfin	Yellowfin	Yellowfin	JN988634
13. Oct18	21.3	Bigeye*	Yellowfin	Yellowfin	JN988633
14. Oct2	22.4	Bigeye*	Yellowfin	Yellowfin	JN988632
15. Oc282	25.5	Yellowfin	Yellowfin	Yellowfin	JN988631
16. Oct19	20.8	Yellowfin	Yellowfin	Yellowfin	JN988630
17. Oc5	17	Yellowfin	Yellowfin	Yellowfin	JN988629
18. Oct16	20.9	Bigeye*	Yellowfin	Yellowfin	JN988628
19. YFT3	29.6	Yellowfin	Yellowfin	Yellowfin	JN988627
20. BET 1	28.8	Bigeye	Bigeye	Bigeye	JN988648
21. Oct12	21.5	Yellowfin	Yellowfin	Yellowfin	JN988626
22. Oct15	22	Yellowfin	Yellowfin	Yellowfin	JN988625
23. N13	20	Yellowfin	Yellowfin	Yellowfin	JN988624
24. N3	17.5	Yellowfin	Yellowfin	Yellowfin	JN988623
25. N17	18.6	Yellowfin	Yellowfin	Yellowfin	JN988622
26. N9	19.5	Yellowfin	Yellowfin	Yellowfin	JN988621
27. Oc19	14.8	Yellowfin	Yellowfin	Yellowfin	JN988620
28. Oc20	18.4	Yellowfin	Yellowfin	Yellowfin	JN988619
29. Oct1	23.2	Yellowfin	Yellowfin	Yellowfin	JN988618
30. N2	19.2	Yellowfin	Yellowfin	Yellowfin	JN988617
31. Oc2650	23.2	Yellowfin*	Bigeye	Bigeye	JN988647
32. N10	18.8	Yellowfin	Yellowfin	Yellowfin	JN988616
33. N1	20.2	Yellowfin	Yellowfin	Yellowfin	JN988615
34. N14	22	Bigeye*	Yellowfin	Yellowfin	JN988614
35. Oct20	22.9	Yellowfin	Yellowfin	Yellowfin	JN988613
36. Oc2611	23	Yellowfin*	Bigeye	Bigeye	JN988646
37. Na23	26	Yellowfin*	Bigeye	Bigeye	JN988645
38. B2	30	Bigeye*	Yellowfin	Yellowfin	JN988612
39. Na24	24.3	Yellowfin	Yellowfin	Yellowfin	JN988611
40. Na26	26.2	Yellowfin	Yellowfin	Yellowfin	JN988610
41. Oc3	15.5	Yellowfin	Yellowfin	Yellowfin	JN988609
42. Oc1	16.6	Yellowfin	Yellowfin	Yellowfin	JN988608
43. Oc8	15.3	Bigeye*	Yellowfin	Yellowfin	JN988607
44. N7	18.7	Yellowfin	Yellowfin	Yellowfin	JN988606
45. Oc11	21.4	Yellowfin	Yellowfin	Yellowfin	JN988605
46. Oc12	13.8	Yellowfin	Yellowfin	Yellowfin	JN988604
47. Oct10	22	Yellowfin	Yellowfin	Yellowfin	JN988603
48. Na11	20	Yellowfin	Yellowfin	Yellowfin	JN988602

* *misidentified.*

## Discussion

Juveniles of yellowfin tuna and bigeye tuna, especially at sizes less than 40 cm FL, are difficult to distinguish using external morphology while DNA-based methods and liver morphology are more reliable for obtaining species identifications (e.g.[8], [16]). With an increasing catch of tuna juveniles, accurate species-level catch data are necessary to determine reproductive activity and to clarify species distributions for fisheries conservation and management [16].

Tuna species can be identified using several genetic markers developed in population-based studies. However, misidentification can occur if the genetic marker is not appropriate for species discrimination [9]. For instance, certain nuclear genetic markers cannot distinguish between Atlantic and Pacific bluefin tuna [17]. Further, genetic markers with low genetic variability, such as mtDNA CO1, infer low genetic distance among *T. albacares*, *T. atlanticus* and *T. tonggol* and prove limited use in differentiation between these species [18].There is therefore a need to consider several premises before attempting the identification of tuna species using mitochondrial genetic markers. Validation of the genetic marker is even more critical due to the observed introgression in some *Thunnus* species [17]. Recently, the mitochondrial DNA control region has been demonstrated to accurately discriminate all species in the genus *Thunnus* [9].

Here, the use of traditional morphological and meristic characters resulted in misidentification of juvenile tuna about 27% of the time. Alternatively, mtDNA CR D-loop data was highly accurate at discriminating juveniles of yellowfin and bigeye tuna with an unambiguous separation between species of 100%. Furthermore, differentiation of the two juveniles liver morphology using the right-lobe liver criterion [12] was confirmed by genetic data showing 1∶1 correspondence; 5 samples (10%) bigeye tunas and 43 samples (90%) yellowfin tunas. This result suggests that liver phenotypes can be a powerful identification tool for fisheries managers on board ships, in the marketplace or in landing sites to provide fast and reliable species identification. Employment of this technique can give cheap means to obtain statistical data on the size of juvenile fishery in the country, which is not available today. Further, should molecular validation be needed for large numbers of tuna specimens, we encourage the development of appropriate restriction enzymes for Restriction Fragment Length Polymorphism analysis or species-specific primers over the slower and more expensive molecular methods applied here.

In this study, the use of the mtDNA CR D-loop coupled with liver phenotype, allows an unequivocal discrimination of the juveniles of yellowfin and bigeye tunas. Proper management can now be achieved once the estimates of these juvenile tunas have been corrected using these two markers.

## Materials and Methods

### Ethics Statement

An institutional review board or equivalent committee is non-existing. Furthermore, the experimental animals used in this research are from landed catch which would mean that the fishes were already dead and no torture was done. These are catch to be vended in the market and there are no strict laws and guidelines relating to their consumption.

### Morpho-meristic Analysis

**Figure 3:**
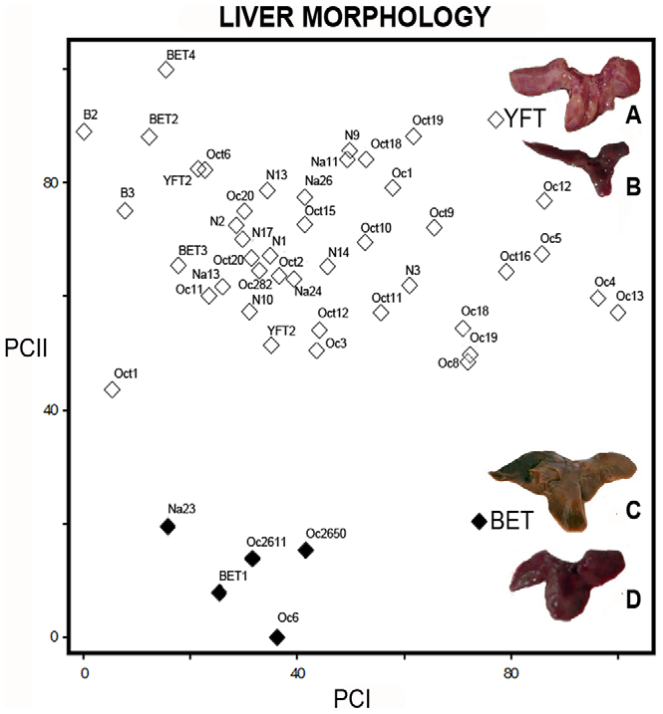
Sampling Site. Map of Southeast Asia showing the fish sampling site (off Southern Iloilo, Philippines) with juveniles of yellowfin and bigeye tunas shown.

Samples of fish (n = 48) taken from the catch of payao-associated purse seine and ring net sets off the Southern Iloilo Peninsula, Philippines (Fig. 3) ranging from 13 to 31 cm FL were used for analysis.

External characters of body marking and bands, eye diameter and body depth were used to initially identify the tuna juveniles. For fresh yellowfin, mid-lateral band is bright yellow, has dark black back that may be separated from the gold by a thin blue band, fins are yellow to yellowish, anal fin sometimes tinged with silver, and flanks and belly silvery white. Yellowfin also has conspicuous alternating bands forward to below pectoral fin. For bigeye, the mid-lateral band is golden to brassy, has dark black back edged with bright metallic blue line, fins are dusky yellowish with anal fin tinged with silver, caudal fin often dusky black, flanks and belly pearly white. Markings are more common on posterior half of the body with few spots. Moreover, yellowfin has a smaller eye diameter and a shorter body depth compared to bigeye of the same FL [12].

Gill-raker counts on the upper limb and lower limb on the first gill arch were also recorded to examine the differences between species. Previous authors have identified a total of 26–35 and 23–31 gill rakers on the first gill arch for yellowfin and bigeye tunas respectively [19].

### Genetic Analysis

Approximately 1 gram (g) muscle tissue was obtained from the dorsal portion of each fish. Tissue samples were placed in 1.5 mL Eppendorf tubes and were kept frozen at −78°C until use.

DNA extraction protocol was based on the Cetyl trimethylammonium bromide (CTAB) extraction method (Doyle and Doyle, 1987) with modifications (Santos *et al.*, 2010). Frozen tissues samples were rinsed with de-ionized water. Approximately 150 mg of the tissue was sliced using uncontaminated disposable razors. Tissue samples were homogenized and placed in a 1.5 mL tube containing CTAB extraction buffer (600 µL of 2% CTAB pH 8.5, 30 µL Proteinase K). The tubes were then incubated overnight in a 55°C water bath with occasional shaking. After incubation, the samples were spun down for 30 s at 6,000 rpm, and 600 µL ofa chloroform∶ isoamyl (3∶1) solution was added to each tube. Tubes were shaken by hand for 3 min then centrifuged for 5 min at 8,000 rpm. The upper supernatant was then transferred into new, properly labeled 1.5 mL tubes. 600 µL of chloroform∶ isoamyl (3∶1) solution was again added to each tube and the steps following CTAB addition were repeated. 50 µL of 3 M sodium acetate (NAOAc) and 900 µL of 95% ethanol were then added and mixed with the supernate in new tubes. Tubes were shaken by hand for 3 min and placed overnight in −20°C freezer to allow the DNA to precipitate out. After precipitation, the samples were centrifuged at 12,000 rpm for 10 min. The aqueous phase was carefully pipetted out and the DNA pellet was left at the bottom of each tube. The pellets were then rinsed twice with 500 µL 70% ethanol and centrifuged at 12,000 rpm for 5 min. The tubes were then opened and allowed to air dry for 30 min then rehydrated in 300 µL of 1×TE buffer. The DNA extracts were then stored at −20°C until molecular analysis.

The mtDNA CR D-loop was amplified from the genomic DNA using the polymerase chain reaction (PCR) technique and two primers (CB3R420 5′ CCCCCTAACTCCCAAAGCTAGG-3′ and 12Sar430 5′ GCCTGCGGGGCTTTCTAGGGCC3′) primarily designed for tuna under the genus *Euthynnus* but also suggested for use in fish closely related to these genus [6]. PCR was carried out in a final volume of 25 µl, in a reaction mixture containing the following reagents: 11.3 µL ddH2O, 2.5 µL 10× PCR Buffer with 1.5 mM MgCl2, 5.0 µL 2 mM dNTPs, 2.5 µL 10 µM Primer 1, 2.5 µL 10 µM Primer 2, 0.2 µL *Taq* DNA Polymerase and 1 µL of DNA template. Individual tubes were subjected to the following cycling parameters in a PCR machine (Labnet International, Inc.): initial denaturation phase of 5 min at 94°C, followed by 35 amplification cycles, each consisting of 1 min of denaturation at 94°C, 1 min of annealing at 50°C, and 1 min of extension at 72°C. Final extension was set at 72°C for 5 minutes.

Agarose gel electrophoresis was used to confirm the successful DNA amplification before sending samples for DNA sequencing. A 1% agarose gel was made by suspending dry agarose in a buffer solution (1 g of agarose to 100 mL of 1× TAE buffer), boiled for approximately 5 minutes or until agarose was completely dissolved , and then poured into a casting tray and allowed it to cool. During electrophoresis, the gel was submersed in a chamber containing a 1× TE buffer solution. The 2 µL of each PCR product was loaded into individual wells with a 3 µL loading dye. The DNA for analysis was forced through the pores of the gel by the electrical current. Electrode wires were connected to the power supply. Positive (red) and negative (black) were made sure to be properly connected. Under an electrical field, DNA moves to the positive electrode and away from the negative electrode [20]. Electrophoresis was allowed to run for 15 min at 100 volts and was visualized under UV light in a gel documentation system. Unclean DNA samples were sent to Macrogen in Korea for purification and DNA Sequencing using 3730/3730xl DNA Analyzer.

Resulting DNA sequences were edited and aligned using alignment explorer MEGA 4.0 [21] and ClustalX 2.0.11 [22]. Percent homology or percent identity between the two species was obtained using Align software [14]. Evolutionary distances were computed using the Maximum Composite Likelihood method [23] and reported as the number of base substitutions per site. All positions containing gaps and missing data were eliminated (complete deletion option) from the dataset. A neighbor-joining phylogenetic tree was inferred using MEGA4 [21] with 1000 bootstrap probability replicates.

All sequences were deposited in GenBank with Accession Numbers JN988602– JN988644 for *T. albacares* and JN988645–JN988649 for *T. obesus*.

### Liver Morphology Analysis

Whole livers were also investigated for each of the samples. These were photographed using a digital camera and the lobes were measured on the longest axis using the Pixel Caliper (version 1.0, UPV, Iloilo, Philippines). Measurement data of the right, left and middle lobes of juvenile yellowfin and bigeye tunas were then log transformed for PCA using PC-ORD 4.10 [24].

Quantifiable difference in the length of the right lobe and overall texture of the liver was noted for each individual. Assumptions were done using the criteria of Itano (2005) for larger individuals, i.e., the right lobe is longer than round medial and left lobes in yellowfin and three rounded lobes are about equal sizes for bigeye. The texture of the livers was also distinct for larger individuals [12], i.e., bigeye livers have striated ventral surface while yellowfins have smooth, clear lobes, and with no striations.
