# Correction: Discrimination of Juvenile Yellowfin (*Thunnus albacares*) and Bigeye (*T. obesus*) Tunas using Mitochondrial DNA Control Region and Liver Morphology

**DOI:** 10.1371/annotation/204a41f1-b918-463f-b444-00313899c455

**Published:** 2012-05-23

**Authors:** Ivane R. Pedrosa-Gerasmio, Ricardo P. Babaran, Mudjekeewis D. Santos

The order of figures were incorrectly switched. The image that appears as Figure 1 should be Figure 3, the image that appears as Figure 2 should be Figure 1, and the image that is Figure 3 should be Figure 2. The figure legends appear in the correct order. 

